# Intraocular Pressure, Axial Length, and Refractive Changes after Phacoemulsification and Trabeculectomy for Open-Angle Glaucoma

**DOI:** 10.1155/2017/1203269

**Published:** 2017-06-04

**Authors:** Alina Popa-Cherecheanu, Raluca Claudia Iancu, Leopold Schmetterer, Ruxandra Pirvulescu, Valeria Coviltir

**Affiliations:** ^1^Carol Davila University of Medicine and Pharmacy, Dionisie Lupu 37 Street, 020021 Bucharest, Romania; ^2^Emergency University Hospital, Department of Ophthalmology, Splaiul Independentei 169, 050098 Bucharest, Romania; ^3^Department of Clinical Pharmacology, Medical University of Vienna, Waehringer Guertel 18-20, 1090 Vienna, Austria; ^4^Center for Medical Physics and Biomedical Engineering, Medical University of Vienna, Waehringer Guertel 18-20, 1090 Vienna, Austria; ^5^Singapore Eye Research Institute, The Academia, 20 College Road Discovery Tower Level 6, Singapore 169856; ^6^Lee Kong Chian School of Medicine, Nanyang Technological University, Novena Campus, 11 Mandalay Road, Singapore 308232; ^7^Ophthalmological Emergency Hospital, Alexandru Lahovari 1 Square, Bucharest, Romania

## Abstract

**Purpose:**

To compare changes in intraocular pressure (IOP), axial eye length (AEL), and refractive outcome in primary open-angle glaucoma patients undergoing cataract surgery and trabeculectomy in dependence of the sequence of surgeries.

**Materials and Methods:**

We retrospectively analysed 48 eyes. The changes in refraction, intraocular pressure, and axial eye length were analysed after surgery. In group A (21 subjects), phacoemulsification was performed before trabeculectomy, and in group B (27 subjects), trabeculectomy was performed before phacoemulsification with a minimum time span between interventions of 6 months.

**Results:**

The reduction in IOP and the decrease in AEL after trabeculectomy were significant after 6 and 12 months postsurgery (*p* < 0.001 each). The decrease in AEL was 0.42 ± 0.11% at 6 months after surgery and 0.40 ± 0.13% after 12 months from surgery; this decrease in AEL was comparable between the groups. The refractive outcome was significantly different between the groups (group A: 0.35 ± 0.75 dpt, group B: −0.05 ± 0.36 dpt, *p* = 0.018); in group A, trabeculectomy caused a hyperopic shift of 0.34 ± 0.44 dpt (*p* = 0.002) at 12 months postsurgery.

**Conclusion:**

IOP reduction after trabeculectomy causes AEL shortening. The effect on refractive outcome depends on the sequence of surgeries. Better refractive outcome is achieved if phacoemulsification is performed after trabeculectomy.

## 1. Introduction

Glaucoma surgery, including trabeculectomy, is aimed to reduce intraocular pressure (IOP). This decrease in IOP is associated with changes in several ocular factors such as shortening of axial eye length (AEL), change in lamina cribrosa position, and increase in ocular blood flow [1–9]. The amount of changes in the eye will depend on the biomechanical properties of the eyeball and will affect ocular refraction [10–13].

This is relevant when glaucoma surgery and phacoemulsification are done in the same eye. One study compared the eyes that underwent phacoemulsification at least 3 months after trabeculectomy with the eyes that did not undergo glaucoma surgery and reported that the change in IOP is negatively correlated with refractive surprise; a correlation between lower postoperative IOP or greater IOP reduction and the amount of axial length reduction was expected [14]. In addition, a recent study has revealed that refractive outcome is inferior in combined versus subsequent trabeculectomy [15]. On the other hand, a study comparing combined trabectome-cataract surgery with cataract-alone surgery did not find any differences in refractive and visual outcomes and a retrospective analysis showed favourable refractive outcomes in patients undergoing simultaneous cataract extraction with trabeculectomy or glaucoma drainage device surgery [16, 17].

In the present analysis, we retrospectively compared the IOP, AEL, and refractive outcome of glaucoma patients undergoing phacoemulsification before trabeculectomy with patients undergoing trabeculectomy after phacoemulsification. This was done to test the hypothesis that there is a significant difference in refractive surprise in the groups, related to the changes in IOP and AEL.

## 2. Materials and Methods

### 2.1. Subjects and Inclusion Criteria

The present analysis stems from retrospectively analysed data of patients that were examined at the Department of Ophthalmology, Emergency University Hospital, Bucharest. Permission by the Ethics Committee of the Emergency University Hospital, Bucharest, was obtained. All subjects gave written informed consent. Patients with primary open-angle glaucoma (POAG) who had undergone both trabeculectomy and phacoemulsification were included in this analysis. The diagnosis of POAG was based on glaucomatous optic neuropathy (cupping, narrowing of the neuroretinal rim mainly at the inferior and superior poles, retinal nerve fibre layer focal defects) associated with characteristic glaucomatous visual field changes. Open angle was verified with gonioscopy, and exclusion of secondary glaucoma was mandatory. All patients had undergone trabeculectomy based on their IOP values, which were not well controlled with medication. A total of 48 eyes of 48 patients were included in this analysis. Of these patients, 21 had phacoemulsification before trabeculectomy (group A) and 27 patients had trabeculectomy before phacoemulsification (group B). The minimum time span between these interventions was 6 months.

### 2.2. Surgical Procedures and Measurements

All subjects were operated by experienced consultant ophthalmologists at the Department of Ophthalmology, Emergency University Hospital, Bucharest. Subjects underwent trabeculectomy augmented with 5-FU standardized with a limbus-based flap at the 12 o'clock position. A precut Weck-Cel sponge was soaked in 50 mg/ml 5-FU and placed between the conjunctiva and sclera. The area was then irrigated with saline solution. A rectangular scleral flap measuring 4 × 4 mm was dissected anteriorly, and a superior iridectomy was performed. The scleral flap was closed with nylon sutures. Phacoemulsification was done in mydriasis under topical anaesthesia with oxybuprocaine 1%. The procedure included a temporal clear corneal incision and implantation of a foldable posterior chamber intraocular lens into the capsular bag. In all cases, a monofocal lens was used. Saline solution was used for irrigation, and an ophthalmic viscoelastic device was also used.

Measurements of AEL were performed using ultrasound immersion biometry (OcuScan, Alcon Laboratories, Fort Worth, Texas). An automatic refractor was used to determine refraction status of the patients (Humphrey Zeiss 5015 Auto Refractor Keratometer, Carl Zeiss Meditec, California). Measurement of IOP was performed using slit lamp-mounted Goldmann applanation tonometry. All measurements were done before trabeculectomy (baseline) as well as at 6 and 12 months after trabeculectomy. The final refractive outcome was compared after both types of surgery had been completed.

### 2.3. Statistical Analysis

Data are presented as means ± SD. In addition, % change over baseline was calculated for all outcome parameters. AEL and IOP data were analysed using a 2-way repeated measures ANOVA using absolute data. Comparison of the change in AEL and the change in IOP was done using linear correlation analysis. To avoid the use of dependent data in correlation analysis, both time points were calculated independently. Refractive outcome between the groups was compared using non-paired *t*-test. The refractive outcome before and after trabeculectomy in group A was compared using a paired *t*-test. A *p* < 0.05 was considered statistically significant. All statistical analysis was performed using SPSS Statistics (StatSoft Inc., Version 6.0, Tulsa, Oklahoma).

## 3. Results

The demographic and clinical data of the 48 analysed subjects before any surgical intervention are presented in Table 1.

**Figure 1:**
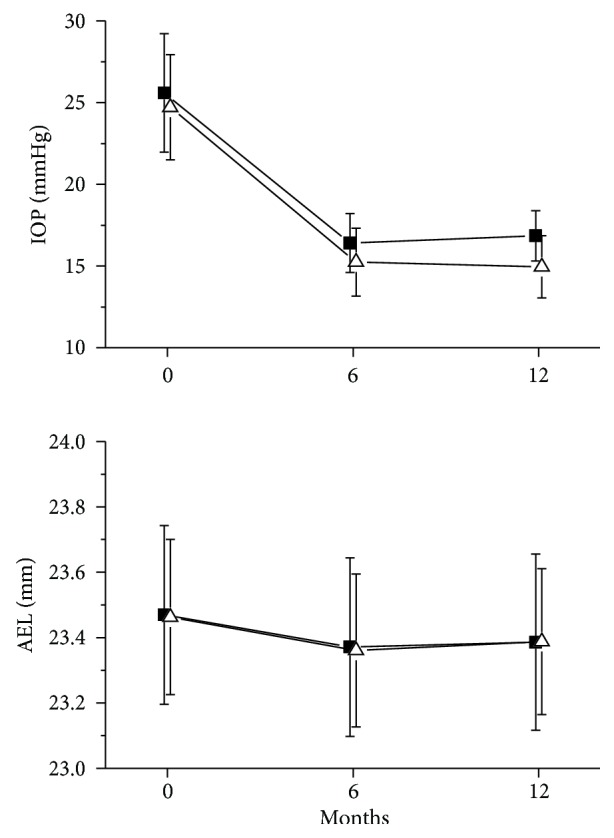
Effect of trabeculectomy on intraocular pressure (IOP) and axial eye length (AEL) in patients undergoing phacoemulsification before trabeculectomy (group A, *n* = 21, open up triangles) and in patients undergoing trabeculectomy before phacoemulsification (group B, *n* = 27, solid squares). Data are presented as means ± SD.

After trabeculectomy, IOP was significantly reduced by 35.6 ± 8.2% and 35.1 ± 8.7% (Figure 1, *p* < 0.001) at 6 and 12 months, respectively.

This reduction in IOP was not significantly different between the groups (Figure 1, *p* = 0.47). In parallel with the decline in IOP, we observed a decrease in AEL. The decrease in AEL was 0.42 ± 0.11% at six months after surgery and 0.40 ± 0.13% after 12 months from surgery (Figure 1, *p* < 0.001). This decrease in AEL was again comparable between the groups (Figure 1, *p* = 0.59).

**Figure 2:**
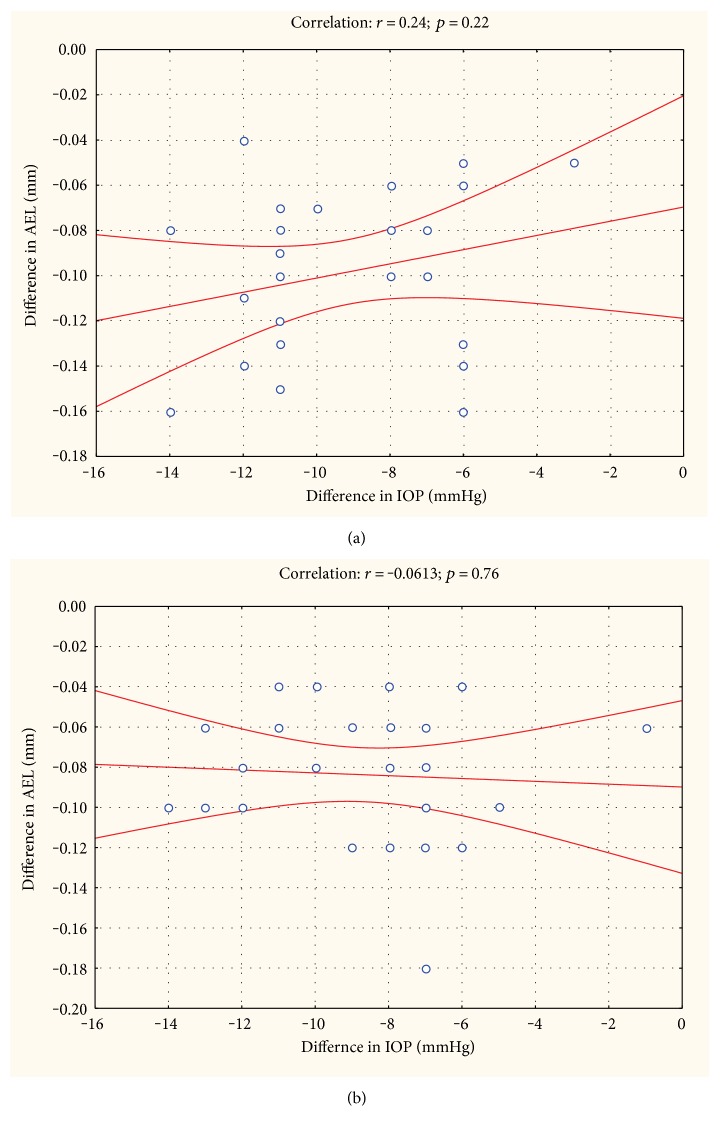
Correlation between changes in intraocular pressure (IOP) and axial eye length (AEL). Data are separately presented for 6 months after surgery (a) and for 12 months after surgery (b). The correlation line and the 95% confidence interval are shown.

Interestingly, no correlation existed between changes in IOP and changes in AEL at either 6 or 12 months (Figure 2).

**Figure 3:**
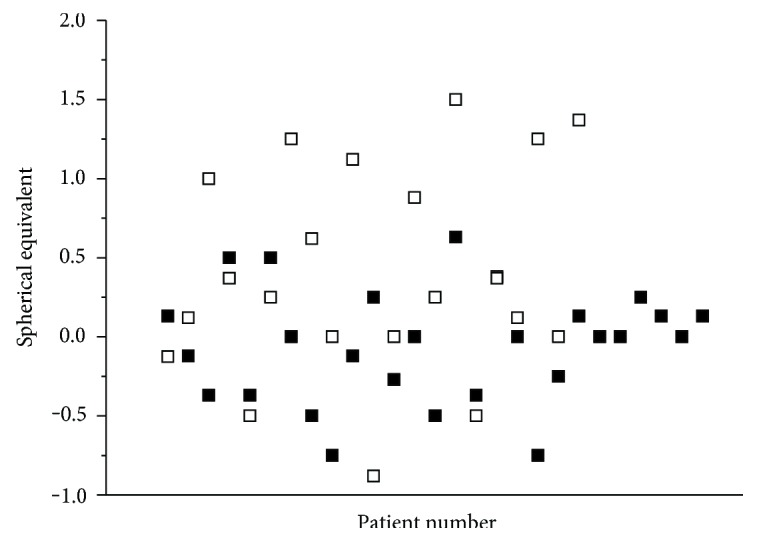
Refractive outcome in patients undergoing phacoemulsification before trabeculectomy (group A, *n* = 21, open squares) and in patients undergoing trabeculectomy before phacoemulsification (group B, *n* = 27, solid squares). A significant difference was observed in spherical equivalent between the groups (*p* = 0.018).

A scatterplot of refractive outcome is presented in Figure 3. Whereas spherical equivalent was only −0.05 ± 0.36 dpt in group B, it was 0.35 ± 0.75 dpt in group A. This difference between the groups was statistically significant at a level of *p* = 0.018.

In group A, trabeculectomy caused a hyperopic shift as compared to postcataract surgery values that were obtained before the filtering surgery. This hyperopic shift was 0.34 ± 0.44 dpt and was significant versus pretrabeculectomy values in group A (0.00 ± 0.58 dpt). The significance level for this effect was *p* = 0.002.

## 4. Discussion

It has been reported in several studies that a reduction in IOP after trabeculectomy or other surgical and pharmacological interventions is associated with a reduction in AEL [1, 2, 18, 19]. This is in good agreement with the data of the present study. On the other hand, an increase in eye length and a myopic shift was observed in the eyes with retinal detachment undergoing combined vitrectomy with cataract surgery related to the increase in IOP showing the close relation between IOP and ocular dimensions [20].

Interestingly, the association between changes in IOP and AEL in the present study was not significant. Several previous studies have quantified the change in AEL when IOP was modified. Short-term elevation in IOP in healthy subjects leads to an increase in AEL by exerting negative pressure to the globe [21]. This is in agreement with work from other authors that showed an increase in IOP by darkroom prone provocative test which is associated with eye elongation in the eyes with angle closure [22]. Kim and coworkers [19] used either pharmacological agents or laser iridotomy to reduce IOP and observed a highly significant correlation between IOP reduction and AEL decrease. Comparison was done immediately after IOP reduction to normal levels had been achieved, but the authors did not provide exact time span between the different study visits. The study is in good agreement with data from experiments in which IOP was lowered with oral acetazolamide leading to eye shortening in both healthy subjects and glaucoma patients [23]. Husain and coworkers [2] studied the relation between IOP and AEL as long as 3 years after surgery and observed that the change in IOP was associated with AEL reduction, but association was weak.

Differences in time may play a role in the association between IOP and AEL on the one hand and the relation between AEL and hyperopic shift on the other hand after IOP reduction. With regard to the former relation, the question is to which degree the change in AEL is representative for the change in intraocular volume [24, 25]. In other words, changes in the shape of the eye ball play a critical role, and it is currently unknown whether different procedures to reduce IOP may differently influence eye shape. Moreover, it needs to be considered that the relation between AEL and IOP after trabeculectomy will also be influenced by changes in choroidal thickness [9, 26]. With regard to the relation between change in AEL and hyperopic shift, anterior chamber depth, corneal diameter, lens thickness, and lens position play a role as well [27, 28]. Obviously, measurement errors in AEL could be responsible for the lack of correlation between changes in IOP and AEL in the present study. In this respect, it needs to be considered that in the most recent studies, biometry was performed based on optical low coherence interferometry, which shows better reproducibility and accuracy than immersion-type ultrasonography employed in our cohort of patients [29, 30]. Participants in the present analysis had, however, frequently very dense cataracts that omitted the use of optical technology. Alternatively, interindividual differences in tissue properties may be responsible for the lack of correlation.

The present study has clinical relevance in terms of the sequence of trabeculectomy and cataract surgery in glaucoma patients. To obtain optimal refractive outcomes, it is definitely advisable to perform trabeculectomy prior to cataract surgery. If this is not possible due to medical reasons, such as very dense cataract, the patient should be informed about the potential consequences on refractive outcomes. This may even be more critical in patients that choose for multifocal lenses although the present analysis did not include such cases.

## Figures and Tables

**Table 1:** Demographic and clinical patient data.

	Group 1	Group 2	*p* value
Age (years)	68.2 ± 5.5	73.2 ± 4.2	0.001
IOP (mmHg)	25.6 ± 3.6	24.7 ± 3.2	0.59
SE	0.64 ± 1.68	0.00 ± 0.58	0.11
AEL (mm)	23.5 ± 3.6	23.5 ± 3.2	0.92
